# Smac/DIABLO enhances the therapeutic potential of chemotherapeutic drugs and irradiation, and sensitizes TRAIL-resistant breast cancer cells

**DOI:** 10.1186/1476-4598-7-60

**Published:** 2008-06-30

**Authors:** Tamer E Fandy, Sharmila Shankar, Rakesh K Srivastava

**Affiliations:** 1 Department of Oncology, The Sidney Kimmel Comprehensive Cancer Center at Johns Hopkins, Baltimore, MD 21231, USA; 2 Department of Biochemistry, The University of Texas Health Science Center at Tyler, TX 75708, USA

## Abstract

**Background:**

Drug resistance is a major concern in cancer therapy. Here, we investigate the clinical potential of the second mitochondria-derived activator of caspase (Smac/DIABLO) in enhancing the apoptosis-inducing potential of commonly used anticancer drugs (paclitaxel, doxorubicin, etoposide, tamoxifen), irradiation and TRAIL in breast carcinoma.

**Methods:**

Breast cancer cells were overexpressed with Smac/DIABLO gene (full-length or Δ55 Smac/DIABLO) or treated with Smac/DIABLO peptide to enhance the apoptosis-inducing potential of chemotherapeutic drugs and irradiation, and sensitize TRAIL-resistant cells. Cell viability and apoptosis were measured by XTT assay and DAPI staining, respectively. Protein-protein interaction was determined by immunoprecipitation followed by the Western blot analysis.

**Results:**

Overexpression of Smac/DIABLO gene (full-length or Δ55 Smac/DIABLO) or treatment with Smac/DIABLO peptide enhances apoptosis induced by paclitaxel, doxorubicin, etoposide, tamoxifen, and irradiation in breast cancer cells. Overexpression of Smac/DIABLO resulted in an increased interaction of Smac/DIABLO with IAPs, which correlated with an increase in caspase-3 activity and apoptosis. Furthermore, Smac/DIABLO sensitized TRAIL-resistant breast cancer cell lines to undergo apoptosis through caspase-3 activation. These data suggest that apoptotic events down-stream of mitochondria were intact in TRAIL-resistant cells since ectopic expression of Smac/DIABLO or pretreatment of cells with Smac/DIABLO peptide completely restored TRAIL sensitivity.

**Conclusion:**

The ability of Smac/DIABLO agonists to enhance the apoptosis-inducing potential of chemotherapeutic drugs and irradiation, and sensitize TRAIL-resistant tumor cells suggests that Smac/DIABLO may induce fundamental alterations in cell signaling pathways. Thus, Smac/DIABLO agonists can be used as promising new candidates for cancer treatment by potentiating cytotoxic therapies.

## Background

The family of cysteine proteases known as caspases are the key components of apoptosis or programmed cell death [1]. TRAIL (TNF-related apoptosis-inducing ligand), a member of TNF family, uses caspase activation as a signaling mechanism leading to apoptosis via two distinct pathways, involving either ligation of death receptors at the cell surface in recruitment of certain procaspases or through the mitochondrial pathway with release of apoptogenic factors such as cytochrome c and Smac/DIABLO into the cytosol along with several other factors such as endonuclease G, apoptotic inducing factor (AIF) and Omi/Htr A2, in parallel with the profound loss of mitochondrial membrane potential [2]. A cross talk exists between apoptotic pathways mediated by cell death receptors and mitochondria through the caspase 8-dependent Bid cleavage (a Bcl-2 family protein) [3]. The activation of initiator caspases such as caspase 8 and caspase 9 is thought to irreversibly trigger the caspase cascade, necessitating that caspase activation is tightly regulated by layered control mechanism.

Several endogenous antagonists of caspase activation pathway which lead to dysregulation of their expression or function in cancer cells have been discovered, such alterations include an impaired ability of the cancer cell to undergo apoptosis. The cellular proteins shown to regulate caspase activation and activity are the IAP's (inhibition of apoptosis protein) including cIAP-1, cIAP-2, XIAP and survivin [4]. These proteins are reported to block death receptors and mitochondrial mediated apoptotic pathways by directly inhibiting initiator and effector caspases. Mitochondrial proapoptotic protein Smac/DIABLO is shown to potentiate apoptosis by counteracting the anti apoptotic function of the IAP's. All IAP's contain at least 1, while some contain 3 BIR (baculovirus IAP repeat) domains [4]. XIAP through BIR domains mediate both its inhibiting activity on caspases and the protein-protein interaction with Smac/DIABLO. During apoptosis, the mitochondrial Smac/DIABLO is released into the cytosol and binds to XIAP by which it antagonizes XIAP interaction with caspase 9, thereby promoting the activity of caspase 9, followed by caspase 3 and apoptosis. The N-terminal peptide (AVPIAQK) of Smac/DIABLO can bind across in a surface groove of BIR 3 of XIAP in a mutually exclusive manner with caspase 9. We have recently demonstrated that mitochondrial events are required for TRAIL-induced apoptosis [5]. Ectopic overexpression of Smac/DIABLO completely restored TRAIL sensitivity by negative regulation of caspase cascade through XIAP [5]. Expression of a cytosolic active form of Smac/DIABLO or cell permeable Smac/DIABLO peptide bypassed the Bcl-2 block, which prevented the release of Smac/DIABLO from mitochondria.

The objective of the paper is to examine whether Smac/DIABLO enhances the apoptosis-inducing potential of chemotherapeutic drugs (paclitaxel, tamoxifen and doxorubicin) and irradiation, and sensitizes TRAIL-resistant breast cancer cells. The results demonstrate that Smac/DIABLO gene or cell permeable Smac/DIABLO peptide enhances the apoptosis-inducing potential of chemotherapeutic drugs and irradiation, and sensitizes TRAIL-resistant breast cancer cells to apoptosis. Thus, Smac/DIABLO gene or Smac/DIABLO peptide can be used to enhance the effectiveness of commonly used anticancer drugs, irradiation and TRAIL in breast cancer.

## Results

### Smac/DIABLO peptide enhances antiproliferative and proapoptotic effects of TRAIL in MCF-7 cells, and sensitizes TRAIL-resistant MDA-MB-453 and MDA-MB-468 cells

We have taken two approaches to examine the effects of Smac/DIABLO in breast cancer cells. In first approach, the Smac/DIABLO (Smac/DIABLO N7, H-AVPIAQK-OH) and control peptides were used. In the second approach, cells were transfected with plasmids expressing full length Smac/DIABLO (pCDNA3-Smac/DIABLO-flag), Δ55 Smac/DIABLO (pCDNA3-Δ55 Smac/DIABLO-flag) or neo (pCDNA3-neo-flag). The NH2 terminus of Smac/DIABLO (55 residues containing the MTSs) is removed by proteolysis to generate the mature and functional form (containing 184 amino acid) of the molecule during mitochondrial import. The goal was to increase the amount of Smac in the cytosol either by pharmacological or genetic method.

**Figure 1:**
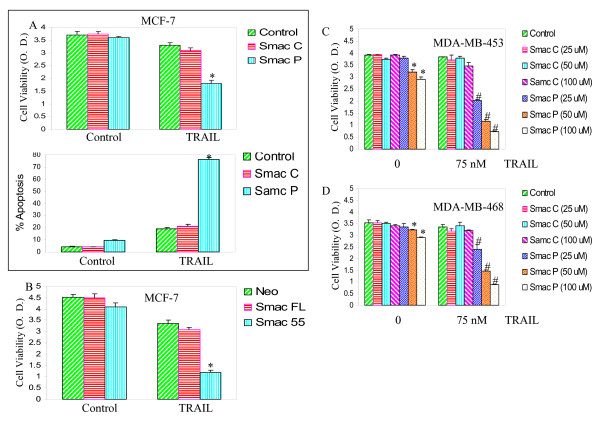
**Interactive effects of Smac/DIABLO with TRAIL on cell viability and apoptosis.** (A), MCF-7 cells were pretreated with either Smac/DIABLO control peptide (25 μM) or Smac/DIABLO N7 peptide (25 μM) for 12 h, followed by treatment with TRAIL (75 nM) for 36 h. Cell viability and apoptosis were measured by XTT assay and DAPI staining, respectively. Data represent mean ± SE. * = significantly different from the respective control at P < 0.05. (B), MCF-7 cells were transiently transfected with plasmids expressing Smac/DIABLO full length, Smac Δ55 or neo for 24 h, and treated with TRAIL (75 nM) for 36 h. Cell viability was measured by XTT assay. Data represent mean ± SE. * = significantly different from the respective control at P < 0.05. Smac C = Smac control peptide, Smac P = Smac N7 peptide. (C), MDA-MB-453 cells were pretreated with various doses of either Smac/DIABLO control peptide (0–100 μM) or Smac/DIABLO N-7 peptide (0–100 μM) for 12 h, followed by treatment with TRAIL (75 nM) for 36 h. Cell viability was measured by XTT assay. Data represent mean ± SE. *, # = significantly different from the respective control at P < 0.05. (D), MDA-MB-468 cells were pretreated with various doses of either Smac/DIABLO control peptide (0–100 μM) or Smac/DIABLO N-7 peptide (0–100 μM) for 12 h, followed by treatment with TRAIL (75 nM) for 36 h. Cell viability was measured by XTT assay. Data represent mean ± SE. *, # = significantly different from the respective control at P < 0.05.

We first measured the effects of Smac/DIABLO on antiproliferative and proapoptotic activity of TRAIL in MCF-7 cells (Fig. 1A). Control Smac/DIABLO peptide (Smac/DIABLO C) had no effect on cell viability and apoptosis. Smac/DIABLO peptide (Smac/DIABLO P) slightly inhibited cell viability and induced apoptosis. TRAIL induced apoptosis in MCF-7 cells pretreated with or without Smac/DIABLO control peptide. The levels of apoptosis by TRAIL in MCF-7 cells pretreated with Smac/DIABLO control peptide was higher than the apoptosis noted in TRAIL-untreated control group. Treatment of MCF-7 cells with Smac/DIABLO peptide increased the effects of TRAIL on cell viability and apoptosis (Fig. 1A). Overexpression of full-length Smac/DIABLO or Δ55 Smac/DIABLO enhanced the antiproliferative effects of TRAIL in MCF-7 cells (Fig. 1B).

We next examined whether Smac/DIABLO peptide can sensitize TRAIL-resistant MDA-MB-453 and MDA-MB-468 breast cancer cells. Smac/DIABLO control peptide had no effect on cell viability (Fig. 1C and 1D). Smac/DIABLO peptide (50 and 100 μM) inhibited cell viability in MDA-MB-453 and MDA-MB-468 cells. Smac/DIABLO peptide (25–100 μM) sensitized TRAIL-resistant MDA-MB-453 and MDA-MB-468 breast cancer cells in a dose-dependent manner. These data suggest that Smac/DIABLO peptide can be used to sensitize TRAIL-resistant breast cancer cells.

### Interactive effects of Smac/DIABLO with chemotherapeutic drugs, irradiation and TRAIL on cell viability, apoptosis and colony formation in breast cancer cells

**Figure 2:**
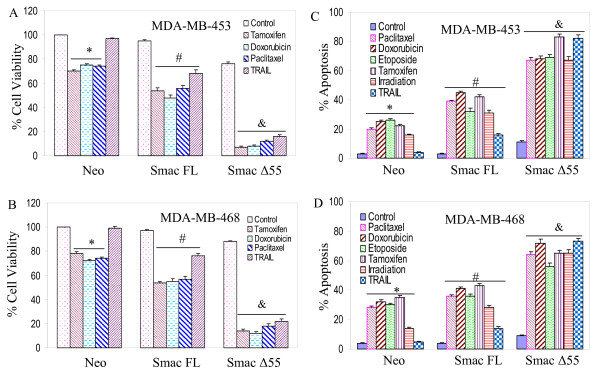
**Effects of Smac/DIABLO with chemotherapeutic drugs or TRAIL on cell viability.** (A), MDA-MB-453 cells were transiently transfected with plasmids expressing Smac/DIABLO full length, Smac Δ55 or neo for 24 h, and treated with tamoxifen (100 nm), doxorubicin (100 nM), paclitaxel (100 nM) or TRAIL (75 nM) for 36 h. Cell viability was measured by XTT assay. Data represent mean ± SE. *, #, & = significantly different from the respective control at P < 0.05. (B), MDA-MB-468 cells were transiently transfected with plasmids expressing Smac/DIABLO full length, Smac Δ55 or neo for 24 h, and treated with tamoxifen (100 nM), doxorubicin (100 nM), paclitaxel (100 nM) or TRAIL (75 nM) for 36 h. Cell viability was measured by XTT assay. Data represent mean ± SE. *, #, & = significantly different from the respective control at P < 0.05. (C), MDA-MB-453 cells were transiently transfected with plasmids expressing Smac/DIABLO full length, Smac Δ55 or neo for 24 h, and treated with paclitaxel (100 nM), doxorubicin (100 nM), etoposide (100 nM), tamoxifen (100 nm), irradiation (5 Gy) or TRAIL (75 nM) for 36 h. Apoptosis was measured by DAPI staining. Data represent mean ± SE. *, #, & = significantly different from the respective control at P < 0.05. (D), MDA-MB-468 cells were transiently transfected with plasmids expressing Smac/DIABLO full length, Smac Δ55 or neo for 24 h, and treated with paclitaxel (100 nM), doxorubicin (100 nM), etoposide (100 nM), tamoxifen (100 nm), irradiation (5 Gy) or TRAIL (75 nM) for 36 h. Apoptosis was measured by DAPI staining. Data represent mean ± SE. *, #, & = significantly different from the respective control at P < 0.05.

In addition to TRAIL, we have also used commonly used anticancer drugs (tamoxifen, doxorubicin and paclitaxel) and irradiation as therapeutic agents. Overexpression of full-length Smac/DIABLO (Smac/DIABLO FL) or mature form of Smac/DIABLO (Smac/DIABLO Δ55) enhanced the inhibitory effects of tamoxifen, doxorubicin, and paclitaxel on cell viability, and sensitized MDA-MB-453 and MDA-MB-468 cells to TRAIL (Fig. 2A and 2B). Furthermore, overexpression of Smac/DIABLO FL or Smac/DIABLO Δ55 enhanced paclitaxel-, doxorubicin-, etoposide, tamoxifen-, and irradiation-induced apoptosis, and sensitized MDA-MB-453 and MDA-MB-468 cells to TRAIL (Fig. 2C and 2D). Similarly, overexpression of Smac/DIABLO FL or Smac/DIABLO Δ55 enhanced paclitaxel-, doxorubicin-, etoposide-, and tamoxifen-induced apoptosis in MCF-7 cells (data not shown). Smac/DIABLO Δ55 was more effective in inhibiting cell viability and promoting apoptosis compared to full length Smac/DIABLO.

**Figure 3:**
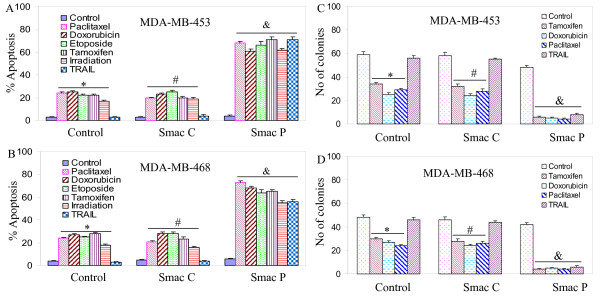
**Interactive effects of Smac/DIABLO peptide with chemotherapeutic drugs or TRAIL on apoptosis and colony formation.** (A), MDA-MB-453 cells were pretreated with either Smac control peptide (25 μM) or Smac N-7 peptide (25 μM) for 12 h, and treated with paclitaxel (100 nM), doxorubicin (100 nM), etoposide (100 nM), tamoxifen (100 nm), irradiation (5 Gy) or TRAIL (75 nM) for 36 h. Apoptosis was measured by DAPI staining. Data represent mean ± SE. *, #, & = significantly different from the respective control at P < 0.05. (B), MDA-MB-468 cells were pretreated with either Smac control peptide (25 μM) or Smac N-7 peptide (25 μM) for 12 h, and treated with paclitaxel (100 nM), doxorubicin (100 nM), etoposide (100 nM), tamoxifen (100 nm), irradiation (5 Gy) or TRAIL (75 nM) for 36 h. Data represent mean ± SE. *, #, & = significantly different from the respective control at P < 0.05. (C), MDA-MB-453 cells were pretreated with either Smac control peptide (25 μM) or Smac N-7 peptide (25 μM) for 12 h, and treated with tamoxifen (100 nm), doxorubicin (100 nM), paclitaxel (100 nM) or TRAIL (75 nM) for 21 days. No. of colonies were determined by soft agar assay. Data represent mean ± SE. *, #, & = significantly different from the respective control at P < 0.05. (D), MDA-MB-468 cells were pretreated with either Smac control peptide (25 μM) or Smac N-7 peptide (25 μM) for 12 h, and treated with tamoxifen (100 nm), doxorubicin (100 nM), paclitaxel (100 nM) or TRAIL (75 nM) for 21 days. No. of colonies were determined by soft agar assay. Data represent mean ± SE. *, #, & = significantly different from the respective control at P < 0.05. represent mean ± SE.

Since overexpression of Smac/DIABLO enhanced the apoptosis-inducing potential of chemotherapeutic drugs and irradiation, and sensitized TRAIL-resistant cells, we next sought to examine the interactive effects of Smac/DIABLO peptide with these agents (Fig. 3A and 3B). Smac control peptide (Smac C) or TRAIL alone had no effect on apoptosis. Paclitaxel, doxorubicin, etoposide, tamoxifen and irradiation induced apoptosis in both MDA-MB-453 and MDA-MB-468 cells. Interestingly, Smac/DIABLO peptide enhanced paclitaxel-, doxorubicin-, etoposide-, tamoxifen-, and irradiation-induced apoptosis, and sensitized TRAIL-resistant MDA-MB-453 and MDA-MB-468 cells to TRAIL. Similarly, Smac/DIABLO peptide enhanced paclitaxel-, doxorubicin-, etoposide-, and tamoxifen-induced apoptosis in MCF-7 cells (data not shown).

Since Smac/DIABLO peptide enhanced the apoptosis-inducing potential of anticancer drugs, and sensitized TRAIL-resistant breast cancer cells, we sought to examine whether they have similar effects on colony formation (Fig. 3C and 3D). Tamoxifen, doxorubicin and paclitaxel inhibited colony formation in both MDA-MD-453 and MDA-MB-468 cells. In contrast, TRAIL or control peptide had no effect on colony formation. Similar to apoptosis, Smac/DIABLO peptide enhanced the inhibitory effects of anticancer drugs on colony formation, and sensitized TRAIL-resistant cells. These data suggest that Smac/DIABLO can be used to enhance the apoptosis-inducing potential of tamoxifen, doxorubicin and paclitaxel, and sensitize TRAIL-resistant breast cancer cells.

### Smac/DIABLO enhances drug-induced apoptosis and sensitizes TRAIL-resistant cells through caspase-3 activation and PARP cleavage

**Figure 4:**
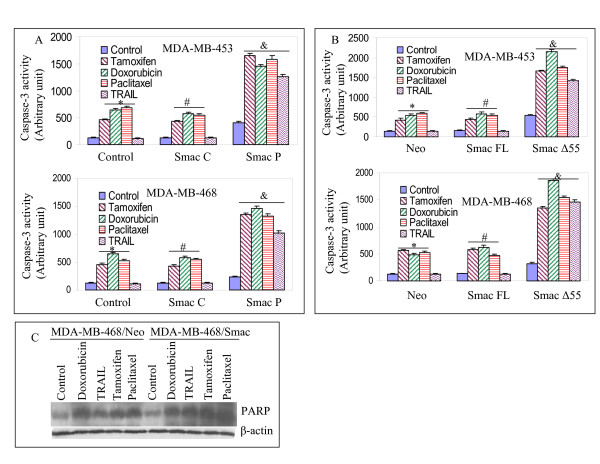
**Interactive effects of Smac/DIABLO peptide with chemotherapeutic drugs or TRAIL on caspase-3 activity and PARP cleavage.** (A), MDA-MB-453 or MDA-MB-468 cells were pretreated with either Smac control peptide (25 μM) or Smac N-7 peptide (25 μM) for 12 h, and treated with tamoxifen (100 nm), doxorubicin (100 nM), paclitaxel (100 nM) or TRAIL (75 nM) for 24 h. Caspase-3 activity was measured as per the manufacturer's instructions (Calbiochem). Data represent mean ± SE. *, #, & = significantly different from the respective control at P < 0.05. (B), MDA-MB-453 or MDA-MB-468 cells were transiently transfected with plasmids expressing Smac/DIABLO full length, Smac Δ55 or neo for 24 h, and treated with tamoxifen (100 nm), doxorubicin (100 nM), paclitaxel (100 nM) or TRAIL (75 nM) for 24 h. Caspase-3 activity was measured as per the manufacturer's instructions (Calbiochem). Data represent mean ± SE. *, #, & = significantly different from the respective control at P < 0.05. (C), MDA-MB-468 cells were transiently transfected with plasmids expressing Smac/DIABLO full length or neo for 24 h, and treated with doxorubicin (100 nM), TRAIL (75 nM), tamoxifen (100 nm) or paclitaxel (100 nM) for 24 h. Western blot analysis was performed to measure the cleavage of PARP. Anti-β-actin antibody was used as a loading control.

Caspase activation appears to be common pathway in apoptosis induced by stress stimuli in many systems [6-8]. Since Smac/DIABLO augments drug-induced apoptosis and sensitizes TRAIL-resistant cells, we sought to examine the mechanism of this interaction by measuring caspase-3 activation. Tamoxifen, doxorubicin and paclitaxel induced caspase-3 activity in both MDA-MD-453 and MDA-MB-468 cells (Fig. 4A). In contrast, TRAIL or control peptide alone had no effect on caspase-3 activity. Treatment of cells with Smac/DIABLO peptide further enhanced drug-induced caspase-3 activation. Furthermore, the combination of Smac/DIABLO and TRAIL resulted in caspase-3 activity in TRAIL-resistant breast cancer cells. Similar effects were obtained with full-length Smac/DIABLO (Smac/DIABLO FL) and Smac/DIABLO Δ55 in both MDA-MD-453 and MDA-MB-468 cells, although Smac/DIABLO Δ55 was more potent than Smac/DIABLO FL (Fig. 4B). These data suggest that Smac/DIABLO enhances the apoptosis-inducing potential of anticancer drugs and sensitizes TRAIL-resistant cells through caspase-3 activation.

Activation of caspase results in cleavage of several substrates such as poly ADP ribose polymerase (PARP) enzyme that can be used as a marker of apoptosis [9,10]. MDA-MB-468 cells were transiently transfected with plasmids expressing Smac/DIABLO (MDA-MB-468/Smac/DIABLO) or neo (MDA-MB-468/neo), and treated with or without doxorubicin, TRAIL, tamoxifen or paclitaxel for 48 h, and cleavage of PARP was determined by the Western blot analysis (Fig. 4C). The antibody recognizes only the cleavage product of PARP. Chemotherapeutic drugs significantly induced PARP cleavage. Overexpression of Smac/DIABLO resulted in enhanced cleavage of PARP in MDA-MB-468/Smac/DIABLO cells treated with doxorubicin, TRAIL, tamoxifen or paclitaxel. These data suggest that Smac/DIABLO enhances the apoptosis-inducing potential of anticancer drugs and sensitizes TRAIL-resistant cells through PARP cleavage.

### Interaction of Smac/DIABLO with IAPs

**Figure 5:**
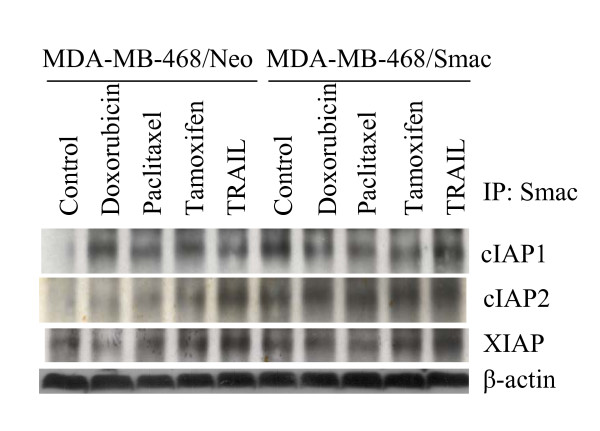
**Interaction of Smac/DIABLO with IAPs.** MDA-MB-468 cells were transiently transfected with plasmids expressing Smac/DIABLO full length (MDA-MB-468/Smac) or neo (MDA-MB-468/neo) gene for 24 h, and treated with doxorubicin (100 nM), paclitaxel (100 nM), tamoxifen (100 nm) or TRAIL (75 nM) for 24 h. Cells were harvested, and immunoprecipitated with anti-Smac antibody. Immunoprecipitated complexes were subjected to SDS-PAGE and immunoblotted with anti-cIAP1, anti-cIAP2 or anti-XIAP antibody. Anti-β-actin antibody was used as a control in whole cell lysate.

The apoptotic death of cells requires proteolytic activation of caspases which are synthesized as latent proenzymes [6,7]. Once activated, caspases cleave a wide range of molecules (e.g. PARP) that eventually result in the dismantlement of cells [11,12]. Active caspases can be specifically inhibited by inhibitors of apoptosis (IAP). IAP antagonists (Smac/DIABLO, Omi/HtrA2 and GSPT1/eRF3) compete with caspases for IAP-binding and consequently relieve caspases and promote cell death. Since Smac/DIABLO augments drug-induced apoptosis, and sensitizes TRAIL-resistant cells, we sought to examine the interactions of Smac/DIABLO with cIAP1, cIAP2 and XIAP. MDA-MB-468/Neo and MDA-MB-468/Smac/DIABLO cells were treated with doxorubicin, paclitaxel, tamaxifen or TRAIL for 24 h (Fig. 5). Cell lysates were immunoprecipitated with anti-Smac/DIABLO antibody, and immunoblotted with anti-cIAP1, cIAP2 or XIAP antibodies. Treatment of MDA-MB-468/neo with chemotherapeutic drugs enhanced the interaction of Smac/DIABLO with IAPs. Interactions of Smac/DIABLO and IAPs were further increased when cells were transfected with Smac/DIABLO. These data suggest that the ability of Smac/DIABLO to enhance drug-induced apoptosis is due to sequestration of IAPs, which, in turn, causes caspase activation and apoptosis.

## Discussion

Our studies provide a rational for the development of combined treatment regimens when Smac/DIABLO agonists enhance the apoptotic response of commonly used chemotherapeutic drugs, irradiation or TRAIL for the treatment of breast cancer. We have shown that Smac/DIABLO enhances the apoptosis-inducing potential of chemotherapeutic drugs (tamoxifen, doxorubicin, or paclitaxel), irradiation and sensitized TRAIL-resistant breast cancer cells *in vitro *by activating caspases-3. Although TRAIL was ineffective alone, Smac/DIABLO sensitizes TRAIL-resistant MDA-MB-468 breast cancer cells.

We have previously documented the role of mitochondria in TRAIL-induced apoptosis [10]. Caspase-8 activation by TRAIL is necessary but not sufficient to induce apoptosis. Crosstalk between the death-receptor and mitochondrial pathways is mediated by caspase-8 cleavage of Bid to tBid [10,13-15]. tBid activates proapoptotic members Bak and Bax to release cytochrome c and Smac/DIABLO from mitochondria [5,16]. We have recently shown that the release of cytochrome c and Smac/DIABLO is differentially regulated by Bax and Bak [5]. In epithelial cells, mitochondria amplify the apoptotic signals leading to activation of caspase-9 followed by caspase-3. The synergistic effects of chemotherapeutic drugs (paclitaxel, vincristine, vinblastine, etoposide, camptothecin and doxorubicin) or irradiation with TRAIL on apoptosis occur through activation of downstream caspase-3, which can be activated by both mitochondria-dependent and -independent pathways [17]. Activation of caspase cleaves several substrates leading to apoptotic cell death.

There are many factors contributing to the resistance to TRAIL. However, it is not clear whether the mechanism of resistance to TRAIL is constitutive or inductive. Several endogenous factors of TRAIL resistance have been proposed; which include (i) low expression of death receptors, (ii) overexpression of cFLIP, proapoptotic members of Bcl-2 family (e.g. Bcl-2 and Bcl-X_L_) and IAPs, (iii) mutations in Bax or/or Bak gene, and (iv) defects in the release in the mitochondrial proteins. Furthermore, the inability of TRAIL to down regulate anti-apoptotic genes and up-regulate pro-apoptotic genes may also contribute to TRAIL sensitivity/resistance. In the present study, we have shown that the exogenous Smac/DIABLO can enhance the apoptotic response of chemotherapeutic drugs and irradiation, and sensitizes TRAIL-resistant cells. In addition, the interaction of Smac/DIABLO with chemotherapeutic drugs or TRAIL on apoptosis occur through enhance binding of IAPs with Smac/DIABLO resulting in an increased activation of caspase-3.

The N-terminus of Smac/DIABLO (55 residues containing the mitochondrial targeting sequences, MTS) is removed by proteolysis to generate the mature and functional form (containing 184 amino acids) of the molecule during mitochondrial import [18,19]. Ectopic overexpression of Smac/DIABLO potentiates epothilone B derivative (BMS)-induced apoptosis [20]. Furthermore, Smac/DIABLO agonists sensitized various tumor cells *in vitro *and *in vivo *for apoptosis induced by death-receptor ligation or cytotoxic drugs [21-23]. Most importantly, Smac/DIABLO peptides strongly enhanced the antitumor activity of TRAIL in an intracranial malignant glioma xenograft model *in vivo *[21]. Complete eradication of established tumors and survival of mice was only achieved upon combined treatment with Smac/DIABLO peptides and TRAIL without detectable toxicity to normal brain tissue. Thus, Smac/DIABLO agonists are promising candidates for cancer therapy by potentiating cytotoxic therapies.

The toxicity of FasL and TNF to non-transformed cells precludes their clinical use [24]. TRAIL, however, causes only minimal toxic effects in normal hepatocytes [25], and the toxic effects can be overcome by simultaneous exposure to the caspase inhibitor, Z-LEHD-FMK [26]. Soluble and native TRAIL has been shown to have no toxicity in rat, mice and nonhuman primates [27,28], suggesting its use as a potential anticancer agent [29]. We have previously shown that most breast cancer cell lines were resistant to TRAIL, and chemotherapeutic drugs (paclitaxel, vincristine, vinblastine, etoposide and camptothecin) or irradiation sensitized TRAIL-resistant cells to undergo apoptosis through upregulation of death receptor DR4 and/or DR5 and activation of caspase-3 [12,30]. In the present study, we have demonstrated that TRAIL-resistant breast cancer cell lines can be sensitized by Smac/DIABLO (peptide or gene).

## Conclusion

We have developed a novel strategy of combining Smac/DIABLO agonists with chemotherapeutic drugs, irradiation or TRAIL for the treatment of human breast cancer. Specifically, Smac/DIABLO increases the apoptosis-inducing potential of chemotherapeutic drugs (paclitaxel, doxorubicin and tamoxifen) and irradiation, and sensitizes TRAIL-resistant cells to undergo apoptosis through enhanced binding of Smac/DIABLO with IAPs and an increased in caspases-3 activity. Furthermore, our studies provide a foundation for the development of combined treatment regimens that would enhance the apoptotic response in both TRAIL-sensitive and TRAIL-resistant cells. Thus, it may be feasible to deliver Smac/DIABLO either through gene therapy or through small molecules/peptides to enhance the clinical applications of commonly used anticancer drugs, irradiation and TRAIL.

## Methods

### Reagents

Anti Smac/DIABLO, cIAP1, cIAP2, XIAP and β-actin antibodies were purchased from Santa Cruz Biotechnology, Inc. (Santa Cruz, CA). Caspase-3 assay kit and antibody against β-actin were purchase from Oncogene Research (Cambridge, MA). Antibodies against caspase-3 and poly ADP ribose polymerase (PARP) were purchased from Biosource International, Inc. (Camarillo, CA). TRAIL was synthesized as described earlier [31]. Enhanced chemiluminescence (ECL) western blot detection reagents were from Amersham Life Sciences Inc. (Arlington Heights, IL). Lipofectamine reagent was from Invitrogen life technologies (Carlsbad, CA).

### Cell culture and transfection

Breast cancer MCF-7, MDA-MB-468 and MDA-MB-453 cells were obtained from the American Type Culture Collection (Manassas, VA). Cells were grown in RPMI 1640 supplemented with D-glucose, HEPES buffer, 2 mM L-glutamine, 1% penicillin-streptomycin mixture, and 10% fetal bovine serum. Cells were grown in tissue culture dishes at 37°C with 5% CO_2_. Cells were transfected with pCDNA3neo-Smac/DIABLO, pCDNA3neo-Δ55 Smac/DIABLO or pCDNA3neo plasmids as we described elsewhere [5].

### Smac/DIABLO peptide

The AVPIAQK sequence (located at the amino terminus after MTS) of Smac/DIABLO is absolutely required for its ability to interact with the baculovirus IAP repeat (BIR3) of XIAP and to promote cytochrome c dependent caspase activation. Smac/DIABLO control peptide (H-MKSDFYF-P-RQIKIWFQNRRMKWKK-OH) and Smac/DIABLO-N7 (H-AVPIAQK-P-RQIKIWFQNRRMKWKK-OH) peptides were used at doses ranging from 25 to 100 μM. The Smac/DIABLO-N7 peptide is modified to be cell permeable by linking the lysine carboxyl terminal to the arginine of *Antennapedia homeodomain *16-mer peptide (underlined) via a proline linker.

### XTT Assay

XTT assays were performed as we described before [32]. In brief, cells (1 × 10^4 ^in 200 μl culture medium per well) were seeded in 96-well plates (flat bottom), and treated with drugs in the presence or absence of TRAIL. Plates were incubated for various time points at 37°C with 5% CO_2_. Before the end of the experiment, 50 μl XTT (sodium 3' [1-(phenylaminocarbonyl)-3,4-tetrazolium]-bis (4-methoxy-6-nitro) benzene sulfonic acid hydrate) labeling mixture (final concentration, 125 μM sodium XTT and 25 μM PMS) per well was added and plates were incubated for additional 4 h at 37°C and 5% CO_2_. The spectrophotometric absorbance of the sample was measured using a microtitre plate (ELISA) reader. The wavelength to measure absorbance of the formazan product was 450 nm, and the reference wavelength was 650 nm.

### Western Blot Analysis

Cells were lysed in a buffer containing 10 mM Tris-HCl (pH 7.6), 150 mM NaCl, 0.5 mM EDTA, 1 mM EGTA, 1% SDS, 1 mM sodium orthovanadate, and a mixture of protease inhibitors (1 mM phenylmethylsulfonyl fluoride, 1 μg/ml pepstatin A, 2 μg/ml aprotinin). Lysates were sonicated for 10 s, centrifuged for 20 min at 10,000 × g and stored at -70°C. Equal amounts of crude proteins were run on 10% SDS-PAGE gels and electrophoretically transferred to nitrocellulose. Nitrocellulose blots were blocked with 6% nonfat dry milk in TBS buffer (20 mM Tris-HCl (pH 7.4), 500 mM NaCl, and 0.01% Tween 20) for 1 hr, washed in TBST three times (10, 5 and 5 min each) and incubated with primary antibody in TBS containing 1% bovine serum albumin overnight at 4°C. Blots were washed three times (10, 5, 5 min each). Immunoreactivity was detected by sequential incubation with horseradish peroxidase-conjugated secondary antibody and ECL reagents.

### Measurement of Apoptosis

Apoptosis was measured by DAPI staining as we described earlier [5].

### Statistical Analyses

For each studied variable, mean and SEM were calculated. Differences between groups were analyzed by one or two way ANOVA (P <0.05).

## Abbreviations

TRAIL: tumor necrosis factor related apoptosis-inducing ligand; Apo-2L: Apo2 ligand; IAP: inhibitors of apoptosis protein; DR: death receptor; DcR: decoy receptor.

## Competing interests

The authors declare that they have no competing interest.

## Authors' contributions

TEF and SS performed the experiments. SS and RKS designed the experiments, supervised the project, and prepare the manuscript. All authors read and approved the final manuscript.
